# Dynamic association of ambient air pollution with incidence and mortality of pulmonary hypertension: A multistate trajectory analysis

**DOI:** 10.1016/j.ecoenv.2023.115126

**Published:** 2023-09-01

**Authors:** Hui Shi, Lan Chen, Shiyu Zhang, Rui Li, Yinglin Wu, Hongtao Zou, Chongjian Wang, Miao Cai, Hualiang Lin

**Affiliations:** a Department of Epidemiology, School of Public Health, Sun Yat-sen University, Guangzhou 510080, China; b Department of Epidemiology and Biostatistics, College of Public Health, Zhengzhou University, Zhengzhou 450001, China

**Keywords:** Ambient air pollution, Pulmonary hypertension, Dynamic association, Multistate analysis

## Abstract

**Background:**

There is little evidence regarding the association between ambient air pollution and incidence and the mortality of pulmonary hypertension (PH).

**Methods:**

We included 494,750 participants at baseline in the UK Biobank study. Exposures to PM_2.5_, PM_10_, NO_2_, and NO_x_ were estimated at geocoded participants' residential addresses, utilizing pollution data provided by UK Department for Environment, Food and Rural Affairs (DEFRA). The outcomes were the incidence and mortality of PH. We used multivariate multistate models to investigate the impacts of various ambient air pollutants on both incidence and mortality of PH.

**Results:**

During a median follow-up of 11.75 years, 2517 participants developed incident PH, and 696 died. We observed that all ambient air pollutants were associated with increased incidence of PH with different magnitudes, with adjusted hazard ratios (HRs) [95% confidence intervals (95% CIs)] for each interquartile range (IQR) increase of 1.73 (1.65, 1.81) for PM_2.5_, 1.70 (1.63, 1.78) for PM_10_, 1.42 (1.37, 1.48) for NO_2_, and 1.35 (1.31, 1.40) for NO_x_. Furthermore, PM_2.5_, PM_10_, NO_2_ and NO_2_ influenced the transition from PH to death, and the corresponding HRs (95% CIs) were 1.35 (1.25, 1.45), 1.31 (1.21, 1.41), 1.28 (1.20, 1.37) and 1.24 (1.17, 1.32), respectively.

**Conclusion:**

The results of our study indicate that exposure to various ambient air pollutants might play key but differential roles in both the incidence and mortality of PH.

## Highlights

• Based on 494,750 participants of UK Biobank datasets.
• Air pollution is dynamically associated with morbidity and mortality in PH.
• Air pollution and smoking synergistically affect the incidence and mortality of PH.

## Graphical Abstract

**Figure:**
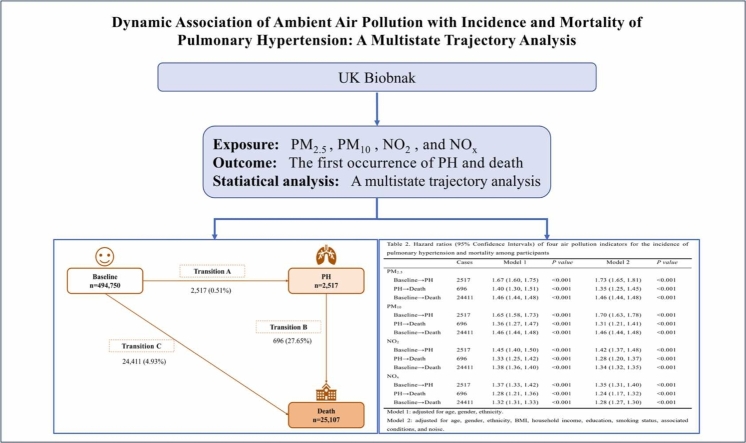

## 1 Introduction

Ambient air pollution is a major environmental threat to human health. It is linked to a wide range of cardiovascular and respiratory diseases, including hypertension, stroke, myocardial infarction (MI), and chronic obstructive pulmonary disease (COPD) (Xie et al., 2021, Ross et al., 2023). However, its effects on some conditions that affect both cardiovascular and pulmonary vascular systems, such as pulmonary hypertension (PH), remain largely unknown.

PH is a fatal, progressive condition of multiple aetiologies, which is defined as a mean pulmonary arterial pressure (mPAP) greater than 20 mmHg (Simonneau et al., 2019). It was estimated that its prevalence was approximately 1% worldwide, making it a global health issue of increasing concern (Hoeper et al., 2016). According to the 6th World Symposium, PH is classified into five different clinical groups, and all these clinical groups may be related to ambient air pollution (Goyanes and Tonelli, 2022). Long-term exposure to particulate matter can cause changes in the pulmonary arteries (Park et al., 2015), and exposure to diesel exhaust can induce PH in mice (Liu et al., 2018). Inhaled air pollutants can cause oxidative stress and systemic inflammation, producing endothelial dysfunction, autonomic nervous system dysregulation, and vasoconstriction (Graber et al., 2019). This demonstrates a biologically plausible relationship between ambient air pollution and PH.

However, large-scale population-based prospective studies exploring the association between ambient air pollution and PH are still lacking. Notably, independent of the underlying disease, the development of PH is associated with a substantially increased risk of death, highlighting the clinical importance of observing the progression of PH as well as clinical endpoints (e.g., death) (Hoeper et al., 2016, Lai et al., 2014). Nevertheless, the influence of air pollution on the dynamic trajectory of PH, especially on the occurrence of PH and deaths, has been less well described.

We therefore assessed the association between ambient air pollution and PH among 494,750 participants from UK Biobank. Importantly, we used a novel multistate model to examine the possibility of distinct impacts of air pollution on the dynamic transitions from baseline to PH and further to death.

## 2 Methods

### 2.1 Study population

The UK Biobank is a prospective cohort study among the general population in the United Kingdom (UK), and the protocol of the study has been previously described (Sudlow et al., 2015). Between 2006 and 2010, more than 0.5 million residents (range 40–69 years of age) were recruited from the UK. All individuals participated in the baseline assessment that included participants' lifestyle, physical measurements, biological samples, and health-related outcomes.

**Fig. 1:**
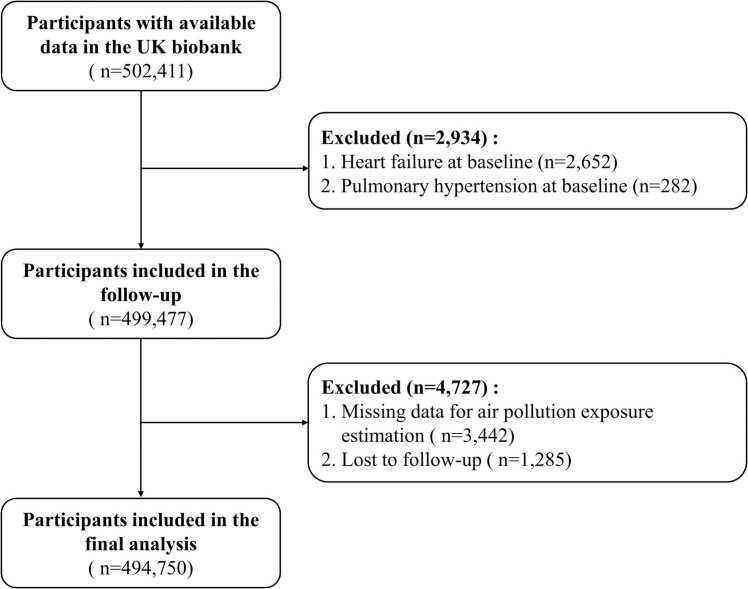
Flowchart for selecting the study population.

Among the 502,411 participants initially recruited for the cohort, those with PH at baseline (n = 282) were excluded. We also excluded those with heart failure at baseline (n = 2652). After excluding individuals without complete ambient air pollution exposure estimates (n = 3442) and those lost to follow-up interviews (n = 1285), the remaining 494,750 participants were finally included in the current analysis (Fig. 1).

The National Research Ethics Service of the National Health Service approved the UK Biobank study (REC reference: 15/NW/0274). The present analysis was carried out using the UK Biobank Resource under Application Number of 69550.

### 2.2 Follow-up for incidence and mortality of PH

The primary outcome of this study was the initial incidence and subsequent mortality of PH after enrolment. Cases of incident PH were ascertained by a combination of hospital admissions and primary care records. Hospital admissions records from Health Episode Statistics in England, Scottish Morbidity Record, and Patient Episode Database for Wales. The data from primary care records provide a detailed record of activities related to symptoms, diagnosis, prescriptions, investigations, and referrals. The date and cause of death were identified from death certificates provided by the National Health Service Information Center and the NHS Central Register Scotland. The International Classification of Diseases (ICD) was used to assign codes to each event. PH was defined by ICD codes (ICD-9: 416.0; ICD-10: I27.0, I27.2) (Clapham et al., 2021). In our study, all-cause mortality excluded accidental deaths (ICD-10: V01-Y89). To mitigate the potential confounding effects of the COVID-19 pandemic, follow-up assessments persisted until the initial PH diagnosis, death, loss to follow-up or the end of study (December 31, 2019), whichever occurred first.

### 2.3 Ambient air pollution exposure estimates

In this study, we assessed the exposure levels of particulate matter with an aerodynamic diameter of 2.5 µm or less (PM_2.5_), particulate matter with an aerodynamic diameter of 10 µm or less (PM_10_), nitrogen dioxide (NO_2_), and nitrogen oxides (NO_x_) by collecting mean annual concentration data for these air pollutants from the UK's Department for Environment, Food and Rural Affairs (DEFRA). DEFRA provides high-resolution near-surface air pollution data for the UK from 2002 to 2020 (Air, 2022), which we utilized to generate annual concentration maps for various air pollutants on a 1 km × 1 km grid via an air dispersion model. This model incorporates data from multiple sources, including measurement data for secondary inorganic aerosols and models for sources such as dust resuspension, from the National Atmospheric Emissions Inventory (Wu et al., 2022). We also calibrated these concentrations with data from background sites in DEFRA's Automatic Urban and Rural Network (Air, 2022).

We utilized a map-based bilinear interpolation algorithm to estimate the air pollutant exposure, which involved calculating the weighted average of the nearest four grids, thereby enhancing the spatial resolution of the target location (Cai et al., 2022). By geocoding participants' residential addresses and corresponding grid cells, we were able to determine the corresponding annual average pollutant concentration (Zou et al., 2023). Furthermore, we collected the residential address histories of participants, including the duration of residence at each location. For those who relocated during the study, we computed the time-weighted average exposure to pollutants, with weights assigned based on the duration of stay at each residence (Huang et al., 2019, Wu et al., 2022). Further details are provided in the Supplementary Materials. Given that baseline year exposure may not provide an accurate representation of long-term exposure levels, we estimated average exposures of the ambient air pollutants from the three years preceding the enrolment to the endpoints of interest (PH or death) or the end follow-up for each participant in the present study.

### 2.4 Assessment of covariates

We conducted a comprehensive assessment of potential covariates based on prior knowledge. A set of variables, including sociodemographic covariates, clinical variables, and noise exposure, were considered. Specifically, sociodemographic covariates included age, gender, ethnicity (white, nonwhite), body mass index (BMI), household income (<£18 000, £18 000–£30 999, £31 000–51 999, £52 000–£100 000, >£100 000), education (having a college or university-level degree or not) (Davies et al., 2016), and smoking status (never, previous, current). Clinical variables including associated conditions and medication use. Associated conditions include left-heart disease, obstructive pulmonary disease, restrictive lung disease, complex congenital heart disease and connective tissue disease (Burger et al., 2021). In addition, self-reported information on medication use, such as lipid- and blood pressure-lowering medications, as well as insulin, was collected. Finally, considering the potential confounding effects of noise exposure (Franklin and Fruin, 2017), we assessed average noise exposure using a model based on Europe's prevalent noise assessment techniques (CNOSSOS-EU) (Kephalopoulos et al., 2014). The World Health Organization suggested that noise pollution be expressed by the 24-hour (daily) sound pressure level (A-weighted sound level in decibels) averaged over a year, i.e., the annual mean 24-hour weighted road traffic noise (Lden) (WHO, 2022).

We constructed a directed acyclic graph (DAG) (www.dagitty.net) to determine which potential covariates should be included in the model (Fig. S1) (Textor et al., 2016). A minimally sufficient adjustment set was selected in the multivariate models, including age, gender, ethnicity, BMI, household income, education, smoking status, associated conditions, and noise.

### 2.5 Statistical analyses

Continuous variables are summarized with descriptive statistics, including the mean (standard deviation [SD]), whereas categorical variables are summarized by the number (percentage). We computed the Spearman correlations between noise and air pollutants.

**Fig. 2:**
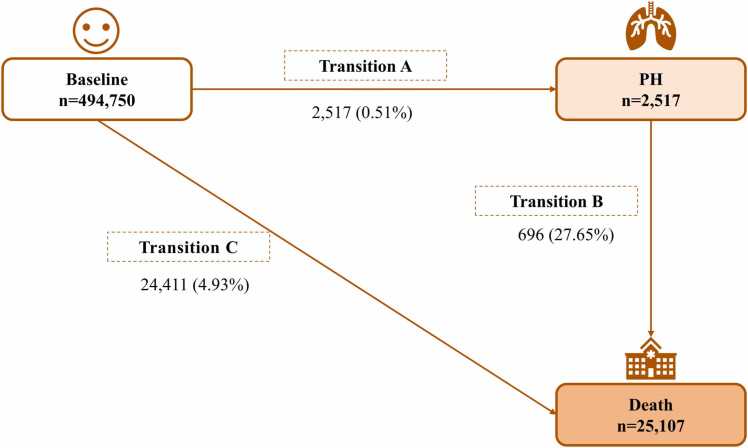
Schematic of multistate model: numbers (percentages) of participants from baseline to pulmonary hypertension (Transition A), pulmonary hypertension subsequent to death (Transition B), and baseline to death (Transition C).

Multistate models provide a flexible framework for modelling the progression of a disease by defining various health states and characterizing the probabilities of transitioning between these states over time (Cannon et al., 2017). It can be employed to examine the progression from a non-disease state to a disease state and ultimately to a death state (Yang et al., 2021). In this study, we constructed a three-state "disease-to-death" Markov multistate model using a clock-forwards approach as the timescale. Specifically, as shown in Fig. 2**,** we divided individuals into three states: baseline (free of PH), PH and death. We considered the severity of the progression of PH and defined irreversible shifts between states (Meyer et al., 2019). The aim of this study was to assess the role of each ambient air pollutant in the temporal course of the disease from baseline (PH-free) to PH and mortality. For 64 participants who entered separate states on the same date, we calculated the supposedly preceding state's entry date as the later state's date minus half a day.

In the current analyses, ambient air pollutants were individually included in the models as continuous variables. Two models were developed. Model 1 adjusted for age, gender, and ethnicity, while Model 2 further adjusted for BMI, household income, education, smoking status, associated conditions, and noise. The latter was selected as the primary model for subsequent analyses. Multiple imputation was performed to impute missing covariate values. Analyses were estimated on each of the imputed data sets, and the results were then combined using the Rubin rule (Zhang, 2016).

We further predicted the probabilities of becoming a person without PH, with PH, and dying from baseline to 1, 3, 5, 10 and 15 years of follow-up. We categorized the levels of five air pollutants into high and low groups based on their respective mean values and then obtained the probabilities of participants exposed to high and low levels of air pollution separately. In the present analysis, all covariates were normalized to the mean level of the UK population.

We performed stratified analyses of potential air pollution effect modification according to smoking status and by introducing cross-product terms. Smoking status was divided into two classes: previous/current smokers and never smokers. There is evidence that tobacco smoke exposure may be a risk factor for PH (Schiess et al., 2010). Furthermore, we conducted stratified analyses to evaluate potential effect modification by age (cut-off 65 years, according to the current retirement age in the UK), gender, education, and income [low (less than £18 000), moderate (£18 000–£100 000), high (greater than £100 000)].

We performed several sensitivity analyses, including (1) analyses for individuals who joined multiple states within the same day that determined the entrance date of the preceding state using various time periods (1, 3, and 5 y); (2) analyses that excluded participants with associated conditions; (3) an analysis that excluded participants without complete data on covariates; (4) further adjustment for medication use at baseline; and (5) the development of 2-pollutant models for each of the 4 air pollutants by integrating 2 air pollutants within the same model. However, PM_10_ and PM_2.5_ showed a strong correlation, as did NO_x_ and NO_2_, so they were not included in the same model. (6) To account for the possibility of spatial misclassification caused by the lack of disease severity information for PH, we added the longitude and latitude of participants as adjusted variables to the model.

Statistical outcomes were presented as hazard ratios (HRs) with 95% confidence intervals (CIs) per change in interquartile range (IQR) pollution exposure. All analyses were performed with R (Version 3.1.0, 2015), and the multistate model was performed using the “mstate” package. Two-sided significance tests were used for all analyses, and statistical significance was set at *P* < 0.05.

## 3 Results

### 3.1 Descriptive results

The final analysis included 494,750 participants, whose baseline characteristics are listed in Table 1. The participants' average age was 56.50 ± 8.09 years, and 54.6% were female. Of all participants, 54.6% were never smokers. Participants with associated conditions accounted for 3.1%. Mean (SD) levels of PM_2.5_, PM_10_, NO_2_, and NO_x_ (the average exposures from three years prior to enrolment until the end of follow-up) were measured as 9.93 ± 1.75, 14.90 ± 2.43, 18.20 ± 5.99, and 27.20 ± 11.00 ug/m^3^, respectively. The mean L_den_ exposure was 56.10 ± 4.30 dB [A]. The Spearman correlation coefficients between air pollution and noise levels ranged from 0.08 to 0.15 (Table S1).

**Table 1:** Baseline characteristics of the study participants.

Variables	Overall (N = 494,750)
***Demographics***	
Age, years	56.50 ± 8.09
Gender, female (%)	269951 (54.6%)
BMI, kg/m2	27.40 ± 4.79
Missing	3003 (0.6%)
Ethnicity (%)	
White	465320 (94.1%)
Nonwhite	26709 (5.4%)
Prefer not to answer	2721 (0.5%)
Household income (%)	
< £18000	95088 (19.2%)
£ 18000 to £ 30999	106494 (21.5%)
£ 31000 to £ 51999	109314 (22.1%)
£ 52000 to £ 100000	85232 (17.2%)
> £100000	22673 (4.6%)
Missing	75949 (15.4%)
Education (%)	
College/university level degree	343710 (69.5%)
Other	141076 (28.5%)
Missing	9964 (2.0%)
Smoking status (%)	
Never	269950 (54.6%)
Previous	169840 (34.3%)
Current	52078 (10.5%)
Prefer not to answer	2013 (0.4%)
Missing	869 (0.2%)
***Clinical characteristics***	
Associated conditions (%)	15387 (3.1%)
Medication at baseline (%)	23084 (4.7%)
***Air and traffic noise exposure***	
PM_2.5_, µg/m^3^	9.93 ± 1.75
PM_10_, µg/m^3^	14.90 ± 2.43
NO_2_, µg/m^3^	18.20 ± 5.99
NO_x_, µg/m^3^	27.20 ± 11.00
L_den_, dB[A]	56.10 ± 4.30
Missing	41060 (8.3%)

Data are mean (SD), or N (%).

BMI, body mass index; PM_2.5_, particulate matter with aerodynamic diameter of less than 2.5 µm; PM_10_, particulate matter with aerodynamic diameter of less than 10 µm; NO_2_, nitrogen dioxide; NO_x_, nitrogen oxides.

Participants were followed up for a mean of 11.75 years (interquartile range: 11.76–12.48 years). Among the participants, 2517 (0.51%) developed PH (Transition A) with a crude incidence rate of 4.31 per 10,000 PYs. A total of 696 (27.65%) incident PH patients died from any cause (Transition B). Additionally, 24,411 (4.93%) participants died without experiencing PH (Transition C) (Fig. 2).

### 3.2 Multistate regression results

Multistate analyses distinguished the roles of four ambient air pollutants in the transitions related to PH (Table 2). After accounting for potential covariates (Model 2), all air pollutants were shown to have statistically significant effects on the transition from baseline to PH (Transition A), with adjusted HRs (95% CIs) per IQR increase of 1.73 (1.65, 1.81) for PM_2.5_, 1.70 (1.63, 1.78) for PM_10_, 1.42 (1.37, 1.48) for NO_2_ and 1.35 (1.31, 1.40) for NO_x_. The associations of PM (PM_2.5_ and PM_10_) with PH incidence were stronger than those of NO_2_ and NO_x_. For Transition B, PM_2.5_, PM_10_, NO_2_ and NO_X_ influenced the transition from PH to death, and the corresponding HRs (95% CIs) were 1.35 (1.25, 1.45), 1.31 (1.21, 1.41), 1.28 (1.20, 1.37) and 1.24 (1.17, 1.32), respectively. In addition, all air pollutants significantly influenced the transition from baseline to death (Transition C).

**Table 2:** Hazard ratios (95% Confidence Intervals) of four air pollution for the incidence of pulmonary hypertension and mortality among participants.

	Cases	Model 1	*P value*	Model 2	*P value*
PM_2.5_					
Baseline→PH	2517	1.67 (1.60, 1.75)	< 0.001	1.73 (1.65, 1.81)	< 0.001
PH→Death	696	1.40 (1.30, 1.51)	< 0.001	1.35 (1.25, 1.45)	< 0.001
Baseline→Death	24411	1.46 (1.44, 1.48)	< 0.001	1.46 (1.44, 1.48)	< 0.001
PM_10_					
Baseline→PH	2517	1.65 (1.58, 1.73)	< 0.001	1.70 (1.63, 1.78)	< 0.001
PH→Death	696	1.36 (1.27, 1.47)	< 0.001	1.31 (1.21, 1.41)	< 0.001
Baseline→Death	24411	1.46 (1.44, 1.48)	< 0.001	1.46 (1.44, 1.48)	< 0.001
NO_2_					
Baseline→PH	2517	1.45 (1.40, 1.50)	< 0.001	1.42 (1.37, 1.48)	< 0.001
PH→Death	696	1.33 (1.25, 1.42)	< 0.001	1.28 (1.20, 1.37)	< 0.001
Baseline→Death	24411	1.38 (1.36, 1.40)	< 0.001	1.34 (1.32, 1.35)	< 0.001
NO_x_					
Baseline→PH	2517	1.37 (1.33, 1.42)	< 0.001	1.35 (1.31, 1.40)	< 0.001
PH→Death	696	1.28 (1.21, 1.36)	< 0.001	1.24 (1.17, 1.32)	< 0.001
Baseline→Death	24411	1.32 (1.31, 1.33)	< 0.001	1.28 (1.27, 1.30)	< 0.001

Model 1: adjusted for age, gender, ethnicity.

Model 2: adjusted for age, gender, ethnicity, BMI, household income, education, smoking status, associated conditions, and noise.

PM_2.5_, particulate matter with aerodynamic diameter of less than 2.5 µm; PM_10_, particulate matter with aerodynamic diameter of less than 10 µm; NO_2_, nitrogen dioxide; NO_x_, nitrogen oxides; PH, pulmonary hypertension.

Furthermore, the impacts of all air pollutants on Transition A were more substantial than those on Transitions B and C. In addition, the effect estimates of all air pollutants on Transition C were slightly stronger than the estimates for Transition B (Table 2).

### 3.3 Prediction based on the multistate model

Based on the results of the multistate model, the estimated transition probabilities from baseline could be calculated for participants. We obtained transition probabilities from baseline to 1, 3, 5, 10 and 15 years of follow-up for participants exposed to high and low air pollutant levels for the three statuses. Table S2 shows the transition probability matrix across statuses, and Fig. S2 shows estimates of stacked transition probabilities.

### 3.4 Stratified analyses

**Fig. 3:**
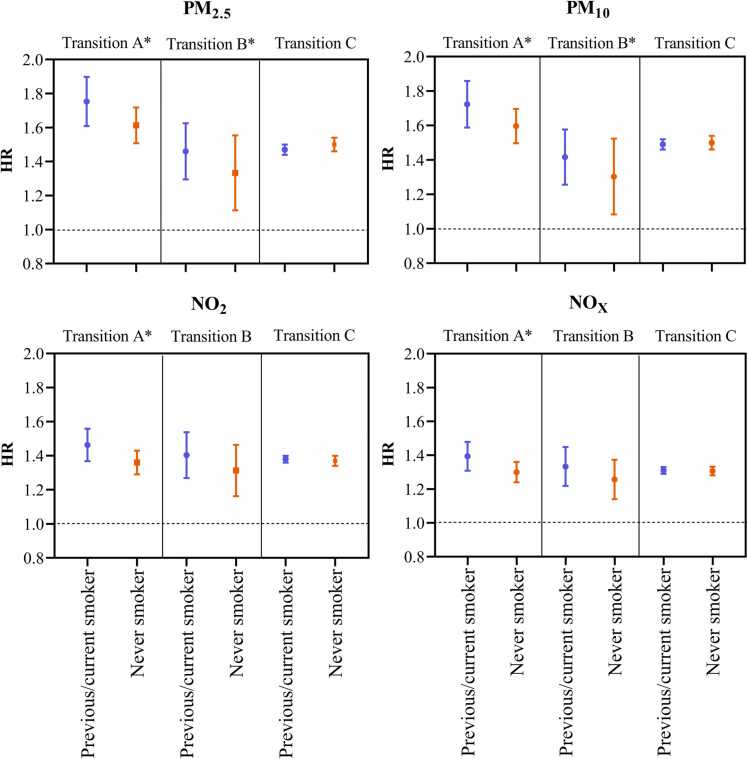
Associations of air pollutions (PM_2.5_, PM_10_, NO_2_, and NO_x_) and three transitions in stratified analyses by smoking statues. Associations are expressed per IQR increase. HR, hazard ratio, PM_2.5_, particulate matter with aerodynamic diameter of less than 2.5 µm; PM_10_, particulate matter with aerodynamic diameter of less than 10 µm; NO_2_, nitrogen dioxide; NO_x_, nitrogen oxides. * , the interaction term was significant (P < 0.05).

Associations between all air pollutants and the incidence of PH may be modified by smoking status. At the same time, smoking status was found to modify the association of particulate matter (PM_2.5_ and PM_10_) with mortality due to PH (Fig. 3). Analyses stratified by smoking status showed that the association between air pollutants and Transition A was stronger in previous or current smokers, and the corresponding HRs (95% CIs) were 1.75 (1.61,1.90) for PM_2.5_, 1.72 (1.59,1.86) for PM_10_, 1.46 (1.37,1.56) for NO_2_, and 1.39 (1.31,1.48) for NO_x_. The associations between particulate matter and PH mortality risk appeared to be stronger among previous or current smokers than never smoker, and the corresponding HRs (95% CIs) were 1.45 (1.30, 1.63) for PM_2.5_, 1.41 (1.26, 1.58) for PM_10_. In addition, the associations between air pollution exposure and Transition C were similar for previous or current smokers and those who have never smoked (Table S3).

We also assessed potential effect modification by gender and education on the incidence and mortality of PH (Tables S4-S5; Fig. S3). The findings indicated that the associations between NO_2_ and PH risk were more pronounced among males. Furthermore, in the low-education group, PM_2.5_, PM_10_, NO_2_, and NO_x_ were more strongly associated with PH risk. However, we did not find a significant modification effect of age and income on the incidence and mortality of PH.

### 3.5 Sensitivity analyses

Several prespecified sensitivity analyses were performed. The sensitivity analyses demonstrated that after adjusting for additional time intervals, the association between air pollution and the risk of PH and subsequent mortality remained significant (Table S6). Further excluded persons reporting associated conditions had no appreciable effect on the findings (Table S7). The results remained robust after excluding participants with incomplete covariate data (Table S8). We found that the results remained consistent with the main analysis, even considering the medication use at baseline (Table S9). In the 2-pollutant model, the associations between PM_10_ and PM_2.5_ and various transitions of PH remained significant and stable. Similarly, the associations between NO_2_ and NO _x_ and mortality of PH remained significant, except for those from baseline to PH (Table S10). The baseline addresses of participants in the model were further adjusted in longitude and latitude, resulting in negligible impact on the observed correlations (Table S11).

## 4 Discussion

The results of the present study demonstrated that exposure to ambient air pollutants played a significant role in the progression of PH, with a greater impact on the incidence of PH than mortality. PM_2.5_ and PM_10_ were stronger associated with the incidence of PH and subsequent death compared to other pollutants. Additionally, our study revealed discrepancies in the relationship between air pollution and the incidence and mortality of PH based on subgroups such as gender, smoking, and education, and identified subgroups that require further attention.

Almost all the air pollutants involved in our study were associated with the incidence of PH. Animal models indicate that exposure to air contaminants such as particulate matter and diesel exhaust alters the pulmonary vasculature (Park et al., 2015, Seimetz et al., 2011). The possibility that air pollution may cause PH is supported by the ability of pollution to activate inflammatory, oxidative stress, and coagulation responses either directly or through translocation into the circulation (Ruckerl et al., 2011).

In the current study, PM_2.5_, PM_10_, NO_2_, and NO_x_ were associated with the risk of death after PH. However, there is currently little information on the association between air pollution and PH mortality, and the results have been inconsistent. Recently, a cohort study including 301 patients with idiopathic or heritable pulmonary arterial hypertension (PAH) showed that a higher estimated dose of PM_2.5_ was related to a greater probability of mortality or lung transplant (Sofianopoulou et al., 2019), which is partially consistent with our findings. In contrast to the findings reported above, Katleen Swinnen et al*.* studied 211 patients with PAH and demonstrated that exposure to PM_2.5_, PM_10_, and NO_2_ was not associated with PAH mortality or poor outcomes (Swinnen et al., 2022), potentially due to the small number of participants. However, they discovered a strong relationship between NO_2_ and the European Society of Cardiology (ESC)/European Respiratory Society (ERS) risk score.

The relationship between air pollution and incidence and the mortality of PH may be related to elevations in the transpulmonary gradient or worsened right ventricular function (Goyanes and Tonelli, 2022). Particulate matter (PM) is a complicated mixture of suspended solid particles and liquid droplets and contains a broad range of toxic substances (Zhang et al., 2021, Sanidas et al., 2017). Among them, PM_2.5_ particles are particularly able to completely penetrate the lungs and permeate into the alveolar epithelium due to their small size (Pope et al., 2002). Pathophysiological exposure to PM can cause damage to airway epithelial cells and lung inflammation and produce oxidative stress, all of which increase endothelial dysfunction, an imbalance in the autonomic nervous system, and vasoconstriction (Mazzoli-Rocha et al., 2010, Weichenthal et al., 2013, Liu et al., 2022). Indeed, inflammation and oxidative stress are crucial to the development of PH (Reis et al., 2013), and vasoconstriction is a critical step in the development of PH (Mandegar et al., 2004). In addition, NO_2_ and NO_x_ have been linked to changes in lung function and heart structure and function in numerous studies (Strak et al., 2012, Kwon et al., 2020). According to the Multi-Ethnic Study of Atherosclerosis (MESA) investigation, exposure to increased levels of NO_2_ was associated with increased right ventricular mass and end-diastolic volume (Leary et al., 2014). The current connection between NO_2_, NO_x_ and PH outcomes is therefore not unexpected, given that the prognosis of PH is predominantly determined by the impact of increasing afterload on right cardiac function.

Our stratified analysis suggests that all air pollution and individual smoking may synergistically impact the incidence of PH, while PM and smoking may synergistically affect the mortality of PH. Adult lung function is negatively correlated with smoking (Dockery et al., 1988). The results of our study can be attributed to the heightened susceptibility of individuals who previously or currently smoke to the impact of pollution, as both smoking and exposure to pollution elicit notable inflammatory reactions in the respiratory system, thereby increasing mortality in PH (Guarnieri and Balmes, 2014).

In stratified analyses, we also found that the associations between NO_2_ and PH risk appeared to be stronger among males. This is in accordance with the literature (Hu et al., 2023). The gender disparities in this study may be explained by differences in airway anatomy, hormones, organ size, social roles, and motivation to seek treatment between males and females (Clougherty, 2010, Manisalidis et al., 2020). In general, deaths from air pollution-related health effects are higher among low-educated groups. The current study demonstrated that in the low-education group, PM_2.5_, PM_10_, NO_2_, and NO_x_ were more strongly associated with PH risk. Negative health consequences are not only from exposure to health risks but also from individual sensitivity to risks (Rao et al., 2021). There are evidence that low-education individuals are more susceptible to disease due to poor nutrition, poor adaptive capacity, and other immunological weaknesses (Kan et al., 2008).

In our study, the concentrations of air pollutants were below the European Union air quality limit values for PM_2.5_ [25 μg/m3], PM_10_ [40 μg/m3], NO_2_ [40 μg/m3] and NO_x_ [30 μg/m3] (Brunekreef et al., 2015). However, our research suggests that exposure to air pollution, even at concentrations below the current air quality thresholds in the European Union, was associated with the incidence and mortality of PH. This suggests that the development of more stringent environmental health policies aimed at mitigating air pollution could potentially result in a decrease in the incidence and mortality of PH.

This study's strengths include its longitudinal methodology, large sample size (>0.46 million individuals), high-resolution exposure estimates, and the definition of a causal framework for the association between covariates and exposures and primary outcomes. Furthermore, we considered the potential variation in ambient air pollution levels before and after enrolment as well as the likelihood of changes in exposure levels resulting from residential relocation. This approach enabled a more rational and precise assessment of long-term air pollution exposure. More importantly, this is the first study to our knowledge to investigate the dynamic relationship between exposure to air pollution and the incidence and mortality of PH, and the models were statistically robust.

This study also has several limitations. First, our study did not include all air pollutants, such as ultrafine particles, that have the potential to impact lung function (Leikauf et al., 2020). Second, even if we account for significant confounding variables, residual confounding may still exist (e.g., accessibility of healthcare visits and treatment of PH). Third, PH is a progressive disease, and accurately assessing its severity at diagnosis may be challenging due to the limited data. This indeterminate spatial misclassification may potentially introduce bias into the relationship between air pollution and PH. Nonetheless, our sensitivity analysis demonstrated that the findings remained robust even after adjusting for the spatial coordinates of the participants (i.e., latitude and longitude). In addition, due to the built-in selection bias (healthy volunteer effect) in the UK Biobank, we may have underestimated the incidence of PH and may limit generalizability. Nevertheless, since the healthy volunteer effect does not affect the validity of exposure–outcome relationships (Batty et al., 2020), our findings are still reliable. Finally, our study is based on the Markovian assumption that the memoryless property of a stochastic process. However, other potential time-varying effects, such as age, can be worth investigating in future studies.

## 5 Conclusion

In conclusion, our results have revealed, for the first time, the differential impact of air pollution on the progression from baseline to PH and mortality. These findings significantly enhance our understanding of the potential effects of air pollution on the pulmonary vasculature, providing valuable new data for evaluating the long-term effect of air pollution exposure on PH. Furthermore, formulating policies that aim to improve air quality and reduce exposure to air pollution is crucial, especially for individuals who are highly susceptible.

## Funding

This work was supported by the 10.13039/501100001809National Natural Science Foundation of China (grant numbers 82041021, Hualiang Lin) and the 10.13039/100000865Bill & Melinda Gates Foundation, Seattle, WA (grant number INV-016826).

## CRediT authorship contribution statement

**Hui Shi:** Conceptualization, Methodology, Formal analysis, Writing − original draft. **Lan Chen:** Validation, Writing − review & editing. **Shiyu Zhang:** Software, Data curation. **Rui Li:** Methodology. **Yinglin Wu:** Methodology**,** Software. **Hongtao Zou:** Methodology, Resources**. Chongjian Wang:** Writing − review & editing. **Miao Cai:** Software, Data curation, Validation**. Hualiang Lin:** Conceptualization, Writing − review & editing, Supervision, Project administration.

## Declaration of Competing Interest

The authors declare that they have no known competing financial interests or personal relationships that could have appeared to influence the work reported in this paper.

## Data Availability

The authors do not have permission to share data.
