# Supplementary material for: Dynamic association of ambient air pollution with incidence and mortality of pulmonary hypertension: A multistate trajectory analysis
Source: Ecotoxicol Environ Saf. 2023 Sep 1;262:115126. doi: 10.1016/j.ecoenv.2023.115126 (PMC10443233; doi:10.1016/j.ecoenv.2023.115126)
Supplement: Supplementary file 1 — Supplementary material. [file mmc1.docx]

**Supplemental Material**

Dynamic Association of Ambient Air Pollution with Incidence and Mortality of Pulmonary Hypertension: A Multistate Trajectory Analysis

Hui Shi ^a^, Shiyu Zhang ^a^, Lan Chen ^a^, Yinglin Wu ^a^, Hongtao Zou ^a^, Chongjian Wang ^b^, Rui Li ^a^, Miao Cai ^a^, Hualiang Lin ^a^*

^a^ Department of Epidemiology, School of Public Health, Sun Yat-sen University, Guangzhou 510080, China

^b^ Department of Epidemiology and Biostatistics, College of Public Health, Zhengzhou University, Zhengzhou 450001, China

* Corresponding author

**Method for estimating air pollution exposure**

In summary, in this study, we determined the exposure levels of PM_2.5_, PM_10_, NO_2_, and NO_x_ from high-resolution air pollution data from the UK Department for Environment, Food and Rural Affairs (DEFRA). Annual pollution maps at 1×1 km resolution was generated using an air dispersion model (Pollution Climate Mapping) and calibrated using monitoring data from background stations in the Defra automated urban and rural network.

Based on this, we used the bilinear interpolation algorithm to estimate pollutant exposure, and subsequently, to achieve more precise long-term exposure estimates, we calculated time weighted average pollutant exposure for participants who changed addresses based on residence history and, where possible, set the average pollutant exposure for each participant from three years prior to baseline to the time of PH onset or death or the end of follow-up. This is particularly important in multistate models, as precise air pollution exposure for participants can be incorporated at different stages. **The specific methods for exposure estimation are as follows:**

**1. Bilinear interpolation algorithm to estimate pollutant exposure**

Bilinear interpolation refers to the process of performing linear interpolation in two axes or directions. The underlying principle involves defining a set of data coordinates, denoted as (x_k_, y_k_), where k=1,2, which specifies the position of the points Q_11_, Q_21_, Q_12_, and Q_22_. By applying the bilinear interpolation technique, we can identify the point P (x, y) for any given coordinates located between the x_k_ and y_k_ points (Figure 1).


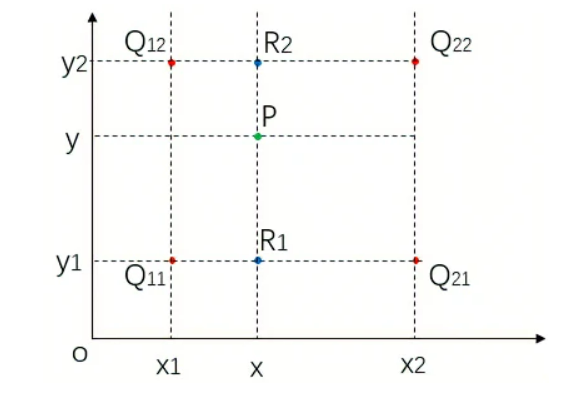
Figure 1. Schematic diagram of bilinear interpolation principle

In our study, we estimated the concentration of air pollution at location A (G(a)) using bilinear interpolation based on maps and the geocoded residential address of each participant. The nearest four grids (G_11_, G_12_, G_21_, and G_22_) were used as the basis, and the weights assigned to each grid (ω_11_, ω_12_, ω_21_, and ω_22_) were determined based on the distance between the participant's residential address and the grids of air pollutants. Specifically, proximity to a grid was positively associated with the weight assigned to that grid, using the following formula ^[1]^:

G(A)=G_11_ω_11_+G_12_ω_12_+G_21_ω_21_+G_22_ω_22_

ω_11_=(x_2_−x_P_) (y_2_−y_P_)/(x_2_−x_1_) (y_2_−y_1_)

ω_12_=(x_2_−x_P_) (y_P_−y_1_)/(x_2_−x_1_) (y_2_−y_1_)

ω_21_=(x_P_−x_1_) (y_2_−y_P_)/(x_2_−x_1_) (y_2_−y_1_)

ω_22_=(x_P_−x_1_) (y_P_−y_1_)/(x_2_−x_1_) (y_2_−y_1_)

**2. A time-weighted average exposure to pollutants**

This method for calculating time-weighted average exposure to contaminants is based on the participant's residential address history, considering the dates the participant lived at each location to determine the weighted the time spent at each residence. The formula can be specified as [2]:

Concentration of air pollution exposure =$\frac{\sum_{i=1}^{j} \left( c_{i}\times d_{i} \right)}{\sum_{i=1}^{j} d_{i}}\left( i=1,2,\ldots,j \right),$

where c_i_ is the annual mean concentration at an address in that year, d_i_ is the number of days at that address in the calendar year, and j is the number of combinations of different addresses and corresponding days in a calendar year. The exposure estimation strategy is presented in Figure 2.

**
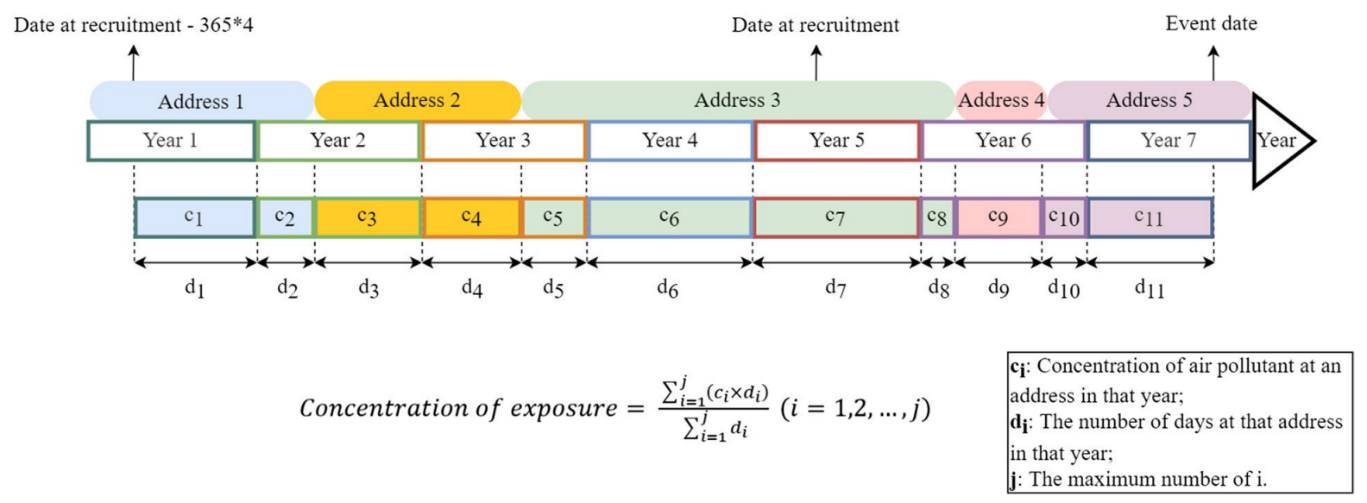
**

Figure 2. The air pollution exposure estimation strategy. The colourful, rounded rectangles represent the participant’s address. The rectangles with colourful borders represent each year during the follow-up period. The colour-filled rectangles with colourful borders represent the concentration of air pollutants at that address in that current year. The fill colour indicates the address, and the border colour indicates the year.

Reference

[1] Cai M, Lin X, Wang X, et al. Long-term exposure to ambient fine particulate matter chemical composition and in-hospital case fatality among patients with stroke in China. The Lancet Regional Health-Western Pacific, 2023: 100679.

[2] Wu Y, Zhang S, Qian SE, et al. Ambient air pollution associated with incidence and dynamic progression of type 2 diabetes: a trajectory analysis of a population-based cohort. BMC Med. 2022;20(1):375.

Table legends:

Table S1. Spearman’s correlation coefficient matrix of the various air pollution and noise variables.

Table S2. Transfer probability matrix for the three states of participants exposed to high and low air pollution.

Table S3. Modification effects of smoking on air pollution and pulmonary hypertension-related outcomes.

Table S4. Modification effects of age and gender on air pollution and pulmonary hypertension -related outcomes.

**Table S5.** Modification effects of income and education on air pollution and pulmonary hypertension -related outcomes.

**Table S6.** Hazard ratios (95% Confidence Intervals) for each transition by ambient air pollutants using the median interval time of the different states in this study to calculate the entering date of the prior state for those who entered different states on the same day.

**Table S7**. Hazard ratios (95% Confidence Intervals) for ambient air pollution indicators for the occurrence of pulmonary hypertension and mortality among participants without any associated conditions.

**Table S8.** Hazard ratios (95% Confidence Intervals) of air pollution indicators for the occurrence of pulmonary hypertension and mortality among participants with complete covariate data.

**Table S9.** Hazard ratios (95% Confidence Intervals) for air pollution indicators on the occurrence of pulmonary hypertension and mortality among participants further adjusted for medication use at baseline.

**Table S10.** Hazard ratios (95% Confidence Intervals) for each transition by ambient air pollutants using 2-pollutant models.

**Table S11.** Hazard ratios (95% Confidence Intervals) of air pollution indicators for the occurrence of pulmonary hypertension and mortality among participants further adjusted for longitude and latitude of the participant's baseline address.

Figure legends:

Figure S1. Directed acyclic graph for the association between air pollution, covariates, and pulmonary hypertension, created with the help of dagitty.net (www.dagitty.net). PH, pulmonary hypertension. Minimally sufficient adjustment sets: age, gender, ethnicity, BMI, household income, education, smoking status, associated conditions, and noise.

Figure S2. Estimation of the stacked transfer probability of three outcomes for participants exposed to high and low air pollution. PM_2.5_, particulate matter with a diameter <2.5 µm; PM_10_, particulate matter with a diameter <10 µm; PM_c_, particulate matter between 2.5 and 10 µm; NO_2_, nitrogen dioxide; NO_x_, nitrogen oxides; PH, pulmonary hypertension.

Figure S3. Associations of air pollution and three transitions in stratified analyses by sociodemographic (age, gender, education, and income). Associations are expressed per IQR increase. HR, hazard ratios, PM_2.5_, particulate matter with a diameter <2.5 µm; PM_10_, particulate matter with a diameter <10 µm; NO_2_, nitrogen dioxide; NO_x_, nitrogen oxides; PH, pulmonary hypertension.

| Table S1. Spearman’s correlation coefficient matrix of the various air pollution and noise variables. | | | | | |
| --- | --- | --- | --- | --- | --- |
|  | PM_2.5_ | PM_10_ | NO_2_ | NO_x_ | Noise |
| PM_2.5_ | 1 | 0.98 | 0.72 | 0.73 | 0.08 |
| PM_10_ | 0.98 | 1 | 0.70 | 0.70 | 0.09 |
| NO_2_ | 0.72 | 0.70 | 1 | 0.99 | 0.15 |
| NO_x_ | 0.73 | 0.70 | 0.99 | 1 | 0.15 |
| Noise | 0.08 | 0.09 | 0.15 | 0.15 | 1 |
| PM_2.5_, particulate matter with a diameter <2.5µm; PM_10_, particulate matter with a diameter <10µm; NO_2_, nitrogen dioxide; NOx, nitrogen oxides. | | | | | |

| Table S2. Transfer probability matrix for the three states of participants exposed to high and low air pollution. | | | | | | | | |
| --- | --- | --- | --- | --- | --- | --- | --- | --- |
|  | PM_2.5_ | | PM_10_ | | NO_2_ | | NO_x_ | |
|  | High  (n=240,749) | Low  (n=254,001) | High (n=238,355) | Low  (n=256,395) | High  (n=222,359) | Low (n=272,391) | High  (n=209,128) | Low  (n=285,622) |
| 1-year follow up |  |  |  |  |  |  |  |  |
| Baseline | 99.67% | 99.71% | 99.69% | 99.70% | 99.68% | 99.70% | 99.67% | 99.70% |
| PH | 0.02% | 0.01% | 0.02% | 0.01% | 0.01% | 0.02% | 0.02% | 0.02% |
| Death | 0.31% | 0.28% | 0.38% | 0.29% | 0.31% | 0.28% | 0.31% | 0.28% |
| 3-year follow up |  |  |  |  |  |  |  |  |
| Baseline | 98.51% | 98.74% | 98.51% | 98.68% | 98.42% | 98.69% | 98.43% | 98.70% |
| PH | 0.09% | 0.07% | 0.09% | 0.07% | 0.09% | 0.07% | 0.09% | 0.07% |
| Death | 1.40% | 1.19% | 1.40% | 1.25% | 1.49% | 1.24% | 1.48% | 1.23% |
| 5-year follow up |  |  |  |  |  |  |  |  |
| Baseline | 96.88% | 97.51% | 97.00% | 97.25% | 96.89% | 97.43% | 96.89% | 97.43% |
| PH | 0.19% | 0.13% | 0.18% | 0.13% | 0.16% | 0.13% | 0.18% | 0.14% |
| Death | 2.93% | 2.36% | 2.82% | 2.62% | 2.95% | 2.44% | 2.93% | 2.43% |
| 10-year follow up |  |  |  |  |  |  |  |  |
| Baseline | 91.64% | 93.36% | 91.66% | 92.76% | 91.62% | 92.80% | 91.65% | 92.66% |
| PH | 0.38% | 0.30% | 0.38% | 0.31% | 0.37% | 0.31% | 0.37% | 0.31% |
| Death | 7.98% | 6.34% | 7.96% | 6.93% | 8.01% | 6.89% | 7.98% | 7.03% |
| 15-year follow up |  |  |  |  |  |  |  |  |
| Baseline | 86.69% | 90.43% | 86.99% | 89.36% | 87.49% | 89.71% | 87.46% | 89.57% |
| PH | 0.54% | 0.41% | 0.54% | 0.41% | 0.53% | 0.40% | 0.53% | 0.40% |
| Death | 12.77% | 9.16% | 12.77% | 10.23% | 11.98% | 9.89% | 12.01% | 10.03% |
| PM_2.5_, particulate matter with a diameter <2.5µm; PM_10_, particulate matter with a diameter <10µm; NO_2_, nitrogen dioxide; NOx, nitrogen oxides. PH, pulmonary hypertension | | | | | | | | |

| Table S3. Modification effects of smoking on air pollution and pulmonary hypertension-related outcomes . | | | | |
| --- | --- | --- | --- | --- |
|  |  | Smoking status | | P for interaction |
|  |  | Previous/current smoker  (n=221,918) | Never smoker  (n=272,832) |  |
| PM_2.5_ | Baseline→PH | 1.75 (1.61,1.90) | 1.61 (1.51, 1.72) | 0.02 |
|  | PH→Death | 1.45 (1.30, 1.63) | 1.32 (1.12,1.56) | 0.001 |
|  | Baseline→Death | 1.47 (1.44, 1.50) | 1.50 (1.46,1.54) | 0.63 |
| PM_10_ | Baseline→PH | 1.72 (1.59,1.86) | 1.59 (1.50, 1.70) | 0.03 |
|  | PH→Death | 1.41 (1.26, 1.58) | 1.29 (1.09,1.53) | 0.04 |
|  | Baseline→Death | 1.49 (1.46, 1.52) | 1.50 (1.46,1.54) | 0.71 |
| NO_2_ | Baseline→PH | 1.46 (1.37,1.56) | 1.36 (1.29, 1.43) | 0.02 |
|  | PH→Death | 1.40 (1.27, 1.54) | 1.39 (1.14,1.41) | 0.29 |
|  | Baseline→Death | 1.38 (1.36, 1.40) | 1.37 (1.34,1.40) | 0.24 |
| NO_x_ | Baseline→PH | 1.39 (1.31,1.48) | 1.30 (1.24, 1.36) | 0.02 |
|  | PH→Death | 1.33 (1.22, 1.45) | 1.28 (1.13,1.36) | 0.22 |
|  | Baseline→Death | 1.31 (1.29, 1.33) | 1.31 (1.28,1.33) | 0.29 |
| PM_2.5_, particulate matter with a diameter <2.5µm; PM_10_, particulate matter with a diameter <10µm; NO_2_, nitrogen dioxide; NO_x,_ nitrogen oxides. PH, pulmonary hypertension | | | | |

| Table S4. Modification effects of age and gender on air pollution and pulmonary hypertension -related outcomes. | | | | | | | |
| --- | --- | --- | --- | --- | --- | --- | --- |
|  |  | Age | | P for interaction | Gender | | P for interaction |
|  |  | < 65 years  (n=93,974) | ≥65 years  (n=400,777) |  | Female  (n=269,951) | Male  (n=224,799) |  |
| PM_2.5_ | Baseline→PH | 1.57 (1.47, 1.68) | 1.69 (1.56, 1.82) | 0.88 | 1.64 (1.52, 1.75) | 1.68 (1.56, 1.81) | 0.13 |
|  | PH→Death | 1.45 (1.27, 1.66) | 1.43 (1.25, 1.64) | 0.06 | 1.37 (1.21, 1.54) | 1.50 (1.28, 1.75) | 0.14 |
|  | Baseline→Death | 1.44 (1.41, 1.47) | 1.43 (1.40, 1.47) | <0.001 | 1.48 (1.44, 1.51) | 1.46 (1.42, 1.49) | 0.57 |
| PM_10_ | Baseline→PH | 1.55 (1.45, 1.65) | 1.67 (1.55, 1.80) | 0.78 | 1.60 (1.50, 1.72) | 1.67 (1.56, 1.80) | 0.08 |
|  | PH→Death | 1.45 (1.43, 1.48) | 1.45 (1.41, 1.48) | 0.12 | 1.34 (1.19, 1.52) | 1.42 (1.22, 1.66) | 0.36 |
|  | Baseline→Death | 1.41 (1.23, 1.62) | 1.38 (1.21, 1.58) | <0.001 | 1.49 (1.46, 1.53) | 1.46 (1.43, 1.50) | 0.32 |
| NO_2_ | Baseline→PH | 1.32 (1.25, 1.40) | 1.39 (1.31, 1.49) | 0.98 | 1.36 (1.28, 1.45) | 1.42 (1.34, 1.51) | 0.04 |
|  | PH→Death | 1.35 (1.20, 1.52) | 1.37 (1.22, 1.53) | 0.88 | 1.30 (1.17, 1.44) | 1.41 (1.23, 1.62) | 0.33 |
|  | Baseline→Death | 1.32 (1.30, 1.35) | 1.34 (1.31, 1.37) | <0.001 | 1.38 (1.36, 1.40) | 1.34 (1.31, 1.37) | 0.09 |
| NO_x_ | Baseline→PH | 1.27 (1.21, 1.34) | 1.33 (1.26, 1.41) | 0.78 | 1.31 (1.24, 1.38) | 1.35 (1.28, 1.43) | 0.07 |
|  | PH→Death | 1.30 (1.17, 1.45) | 1.30 (1.18, 1.44) | 0.93 | 1.25 (1.13, 1.37) | 1.36 (1.20, 1.54) | 0.31 |
|  | Baseline→Death | 1.27 (1.25, 1.29) | 1.28 (1.26, 1.30) | <0.001 | 1.31 (1.29, 1.33) | 1.28 (1.26, 1.31) | 0.11 |
| PM_2.5_, particulate matter with a diameter <2.5µm; PM_10_, particulate matter with a diameter <10µm; PM_c_, particulate matter between 2.5 and 10 µm; NO_2_, nitrogen dioxide; NO_x_, nitrogen oxides. PH, pulmonary hypertension | | | | | | | |

| Table S5. Modification effects of income and education on air pollution and pulmonary hypertension -related outcomes. | | | | | | | | | |
| --- | --- | --- | --- | --- | --- | --- | --- | --- | --- |
|  |  | Income | | | P^a^ for interaction | P^b^ for interaction | Education | | P for interaction |
|  |  | Low  (n=95,088) | Moderate  (n=301,040) | High  (n=98,622) |  |  | Collage  (n=158,916) | Others  (n=335,834) |  |
| PM_2.5_ | Baseline→PH | 1.60(1.48, 1.73) | 1.71(1.57, 1.86) | 1.66(1.49, 1.86) | 0.55 | 0.52 | 1.51 (1.36, 1.67) | 1.72 (1.62, 1.82) | 0.01 |
|  | PH→Death | 1.43(1.22, 1.66) | 1.36(1.16, 1.59) | 1.66(1.36, 2.00) | 0.13 | 0.25 | 1.36 (1.10, 1.68) | 1.46 (1.31, 1.62) | 0.32 |
|  | Baseline→Death | 1.49(1.46, 1.52) | 1.47(1.43, 1.51) | 1.42(1.37, 1.47) | 0.007 | 0.002 | 1.39 (1.35, 1.44) | 1.50 (1.48, 1.53) | <0.001 |
| PM_10_ | Baseline→PH | 1.57(1.45, 1.69) | 1.71(1.58, 1.86) | 1.63(1.47, 1.81) | 0.33 | 0.52 | 1.48 (1.34, 1.63) | 1.70 (1.60, 1.80) | 0.01 |
|  | PH→Death | 1.38(1.18, 1.60) | 1.32(1.12, 1.55) | 1.62(1.32, 1.98) | 0.13 | 0.22 | 1.32 (1.07, 1.63) | 1.42 (1.27, 1.58) | 0.31 |
|  | Baseline→Death | 1.49(1.46, 1.53) | 1.50(1.46, 1.54) | 1.42(1.38, 1.48) | 0.001 | 0.002 | 1.39 (1.35, 1.43) | 1.53 (1.50, 1.55) | <0.001 |
| NO_2_ | Baseline→PH | 1.32(1.24, 1.41) | 1.45(1.35, 1.56) | 1.42 (1.30, 1.55) | 0.58 | 0.16 | 1.26 (1.16, 1.37) | 1.44 (1.37, 1.52) | 0.002 |
|  | PH→Death | 1.35(1.19, 1.54) | 1.37(1.19, 1.57) | 1.42(1.17, 1.73) | 0.81 | 0.11 | 1.38 (1.16, 1.66) | 1.35 (1.23, 1.48) | 0.81 |
|  | Baseline→Death | 1.36(1.33, 1.39) | 1.39(1.35, 1.42) | 1.34(1.30, 1.37) | 0.001 | 0.81 | 1.26 (1.23, 1.30) | 1.42 (1.39, 1.44) | <0.001 |
| NO_X_ | Baseline→PH | 1.28(1.20, 1.36) | 1.37(1.29, 1.47) | 1.34(1.24, 1.45) | 0.47 | 0.29 | 1.22 (1.13, 1.32) | 1.38 (1.32, 1.44) | 0.003 |
|  | PH→Death | 1.30(1.16, 1.46) | 1.3 (1.15, 1.47) | 1.38(1.16, 1.65) | 0.62 | 0.66 | 1.31 (1.11, 1.54) | 1.30 (1.20, 1.41) | 0.71 |
|  | Baseline→Death | 1.30(1.28, 1.32) | 1.32(1.29, 1.35) | 1.27(1.24, 1.31) | <0.001 | 0.06 | 1.22 (1.19, 1.24) | 1.35 (1.33, 1.37) | <0.001 |
| PM_2.5_, particulate matter with a diameter <2.5µm; PM_10_, particulate matter with a diameter <10µm; PM_c_, particulate matter between 2.5 and 10 µm; NO_2_, nitrogen dioxide; NO_x_, nitrogen oxides. PH, pulmonary hypertension  a, high vs. moderate; b, high vs. low. | | | | | | | | | |

| Table S6. Hazard ratios (95% Confidence Intervals) for each transition by ambient air pollutants using the median interval time of the different states in this study to calculate the entering date of the prior state for those who entered different states on the same day. | | | |
| --- | --- | --- | --- |
|  | Time intervals (1y) HR (95% CI) | Time intervals (3y) HR (95% CI) | Time intervals (5y) HR (95% CI) |
| PM_2.5_ |  |  |  |
| Baseline→PH | 1.66 (1.58, 1.75) | 1.66 (1.58, 1.75) | 1.66 (1.58, 1.75) |
| PH→Death | 1.37 (1.24, 1.51) | 1.35 (1.23, 1.49) | 1.36 (1.24, 1.50) |
| Baseline→Death | 1.46 (1.44, 1.48) | 1.46 (1.44, 1.48) | 1.46 (1.44, 1.48) |
| PM_10_ |  |  |  |
| Baseline→PH | 1.64 (1.56, 1.73) | 1.64 (1.56, 1.73) | 1.64 (1.56, 1.73) |
| PH→Death | 1.33 (1.21, 1.47) | 1.32 (1.19, 1.45) | 1.32 (1.20, 1.46) |
| Baseline→Death | 1.47 (1.45, 1.5) | 1.47 (1.45, 1.50) | 1.47 (1.45, 1.50) |
| NO_2_ |  |  |  |
| Baseline→PH | 1.39 (1.33, 1.46) | 1.39 (1.33, 1.46) | 1.39 (1.33, 1.46) |
| PH→Death | 1.31 (1.20, 1.43) | 1.29 (1.19, 1.41) | 1.29 (1.18, 1.40) |
| Baseline→Death | 1.35 (1.33, 1.37) | 1.35 (1.33, 1.37) | 1.35 (1.33, 1.37) |
| NO_X_ |  |  |  |
| Baseline→PH | 1.33 (1.28, 1.39) | 1.33 (1.28, 1.39) | 1.33 (1.28, 1.39) |
| PH→Death | 1.26 (1.17, 1.36) | 1.25 (1.16, 1.35) | 1.24 (1.15, 1.35) |
| Baseline→Death | 1.29 (1.27, 1.31) | 1.29 (1.27, 1.31) | 1.29 (1.27, 1.31) |
| PM_2.5_, particulate matter with a diameter <2.5µm; PM_10_, particulate matter with a diameter <10µm; NO_2_, nitrogen dioxide; NO_x_, nitrogen oxides. PH, pulmonary hypertension | | | |

| Table S7. Hazard ratios (95% Confidence Intervals) for ambient air pollution indicators for the occurrence of pulmonary hypertension and mortality among participants without any associated conditions | |
| --- | --- |
|  | None associated conditions(n=479,363) |
| PM_2.5_ |  |
| Baseline→PH | 1.68 (1.59, 1.78) |
| PH→Death | 1.41 (1.25, 1.58) |
| Baseline→Death | 1.50 (1.48, 1.53) |
| PM_10_ |  |
| Baseline→PH | 1.65 (1.55, 1.74) |
| PH→Death | 1.34 (1.20, 1.50) |
| Baseline→Death | 1.51 (1.48, 1.53) |
| NO_2_ |  |
| Baseline→PH | 1.41 (1.34, 1.48) |
| PH→Death | 1.36 (1.23, 1.50) |
| Baseline→Death | 1.38 (1.36, 1.40) |
| NO_X_ |  |
| Baseline→PH | 1.37 (1.32, 1.43) |
| PH→Death | 1.29 (1.20, 1.39) |
| Baseline→Death | 1.33 (1.32, 1.35) |
| PM_2.5_, particulate matter with a diameter <2.5µm; PM_10_, particulate matter with a diameter <10µm; NO_2_, nitrogen dioxide; NO_x_, nitrogen oxides. PH, pulmonary hypertension | |

| Table S8. Hazard ratios (95% Confidence Intervals) of ambient air pollution indicators for the occurrence of pulmonary hypertension and mortality among participants with complete covariate data. | |
| --- | --- |
|  | Complete data of covariates (n=393,015) |
| PM_2.5_ |  |
| Baseline→PH | 1.63 (1.53, 1.74) |
| PH→Death | 1.41 (1.23, 1.62) |
| Baseline→Death | 1.47 (1.44, 1.50) |
| PM_10_ |  |
| Baseline→PH | 1.60 (1.50, 1.70) |
| PH→Death | 1.35 (1.18, 1.55) |
| Baseline→Death | 1.48 (1.45, 1.51) |
| NO_2_ |  |
| Baseline→PH | 1.35 (1.28, 1.43) |
| PH→Death | 1.37 (1.22, 1.54) |
| Baseline→Death | 1.34 (1.32, 1.36) |
| NO_X_ |  |
| Baseline→PH | 1.30 (1.24, 1.37) |
| PH→Death | 1.32 (1.18, 1.47) |
| Baseline→Death | 1.28 (1.26, 1.30) |
| PM_2.5_, particulate matter with a diameter <2.5µm; PM_10_, particulate matter with a diameter <10µm; NO_2_, nitrogen dioxide; NO_x_, nitrogen oxides. PH, pulmonary hypertension | |

| Table S9. Hazard ratios (95% Confidence Intervals) for air pollution indicators on the occurrence of pulmonary hypertension and mortality among participants further adjusted for medication use at baseline | |
| --- | --- |
|  | Hazard ratios (95% CIs) |
| PM_2.5_ |  |
| Baseline→PH | 1.66 (1.57, 1.76) |
| PH→Death | 1.39 (1.24, 1.56) |
| Baseline→Death | 1.47 (1.45, 1.50) |
| PM_10_ |  |
| Baseline→PH | 1.63 (1.54, 1.72) |
| PH→Death | 1.34 (1.20, 1.50) |
| Baseline→Death | 1.48 (1.45, 1.50) |
| NO_2_ |  |
| Baseline→PH | 1.38 (1.32, 1.45) |
| PH→Death | 1.34 (1.22, 1.48) |
| Baseline→Death | 1.35 (1.33, 1.37) |
| NO_X_ |  |
| Baseline→PH | 1.32 (1.27, 1.38) |
| PH→Death | 1.29 (1.18, 1.41) |
| Baseline→Death | 1.29 (1.27, 1.31) |
| PM_2.5_, particulate matter with a diameter <2.5µm; PM_10_, particulate matter with a diameter <10µm; NO_2_, nitrogen dioxide; NO_x_, nitrogen oxides. PH, pulmonary hypertension | |

| Table S10. Hazard ratios (95% Confidence Intervals) for each transition by ambient air pollutants using 2-pollutant models | | | | |
| --- | --- | --- | --- | --- |
| Pollutant | Model | HR (95% CI), per IQR increment in pollutant concentration | | |
|  |  | Baseline→PH | PH→Death | Baseline→Death |
| PM_2.5_ | + NO_2_ | 1.78 (1.63, 1.95) | 1.25 (1.04, 1.49) | 1.33 (1.29, 1.37) |
|  | + NO_X_ | 1.85 (1.69, 2.02) | 1.31 (1.10, 1.57) | 1.41 (1.37, 1.45) |
| PM_10_ | + NO_2_ | 1.72 (1.58, 1.88) | 1.14 (0.95, 1.35) | 1.38 (1.34, 1.42) |
|  | + NO_X_ | 1.79 (1.64, 1.96) | 1.19 (1.00, 1.42) | 1.46 (1.42, 1.50) |
| NO_2_ | + PM_2.5_ | 0.94 (0.87, 1.02) | 1.12 (0.95, 1.31) | 1.11 (1.08, 1.14) |
|  | + PM_10_ | 0.96 (0.89, 1.03) | 1.20 (1.03, 1.40) | 1.08 (1.05, 1.10) |
| NO_X_ | + PM_2.5_ | 0.91 (0.84, 0.97) | 1.05 (0.91, 1.22) | 1.03 (1.01, 1.06) |
|  | + PM_10_ | 0.92 (0.86, 0.98) | 1.01 (0.99, 1.03) | 1.12 (0.97, 1.30) |
| PM_2.5_, particulate matter with a diameter <2.5µm; PM_10_, particulate matter with a diameter <10µm; NO_2_, nitrogen dioxide; NO_x_, nitrogen oxides. PH, pulmonary hypertension | | | | |

| Table S11. Hazard ratios (95% Confidence Intervals) of air pollution indicators for the occurrence of pulmonary hypertension and mortality among participants further adjusted for longitude and latitude of the participant's baseline address | |
| --- | --- |
|  | Hazard ratios (95% CIs) |
| PM_2.5_ |  |
| Baseline→PH | 2.77 (2.55, 3.01) |
| PH→Death | 2.93 (2.48, 3.46) |
| Baseline→Death | 3.35 (3.27, 3.43) |
| PM_10_ |  |
| Baseline→PH | 2.38 (2.20, 2.57) |
| PH→Death | 2.32 (1.99, 2.71) |
| Baseline→Death | 2.93 (2.86, 3.00) |
| NO_2_ |  |
| Baseline→PH | 1.38 (1.31, 1.45) |
| PH→Death | 1.48 (1.34, 1.63) |
| Baseline→Death | 1.52 (1.50, 1.54) |
| NO_X_ |  |
| Baseline→PH | 1.32 (1.26, 1.38) |
| PH→Death | 1.41 (1.28, 1.54) |
| Baseline→Death | 1.43 (1.41, 1.45) |
| PM_2.5_, particulate matter with a diameter <2.5µm; PM_10_, particulate matter with a diameter <10µm; NO_2_, nitrogen dioxide; NO_x_, nitrogen oxides. PH, pulmonary hypertension | |


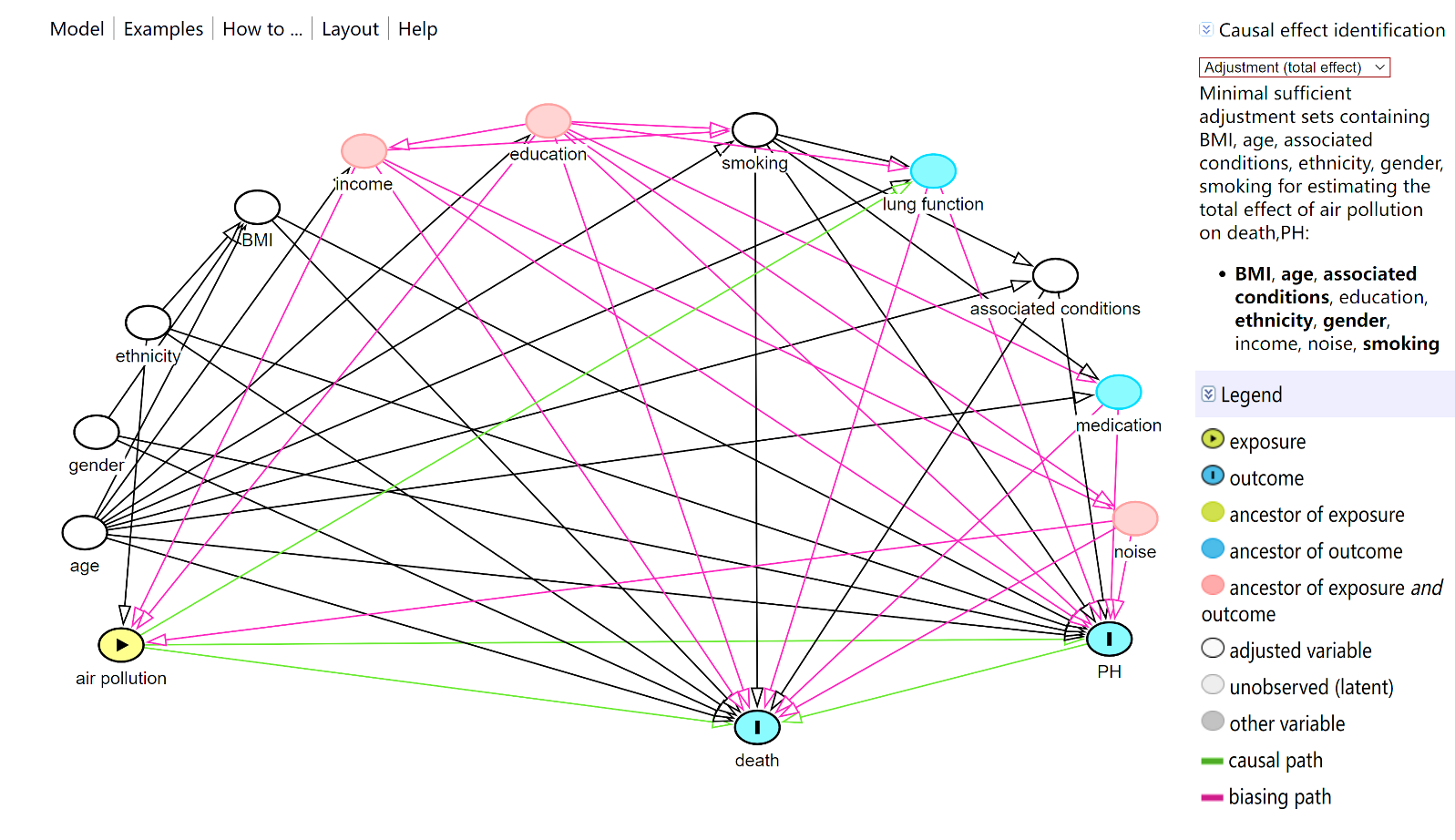


Figure S1. Directed acyclic graph for the association between air pollution, covariates, and pulmonary hypertension, created with the help of dagitty.net (www.dagitty.net). PH, pulmonary hypertension. Minimally sufficient adjustment sets: age, gender, ethnicity, BMI, household income, education, smoking status, associated conditions, and noise.


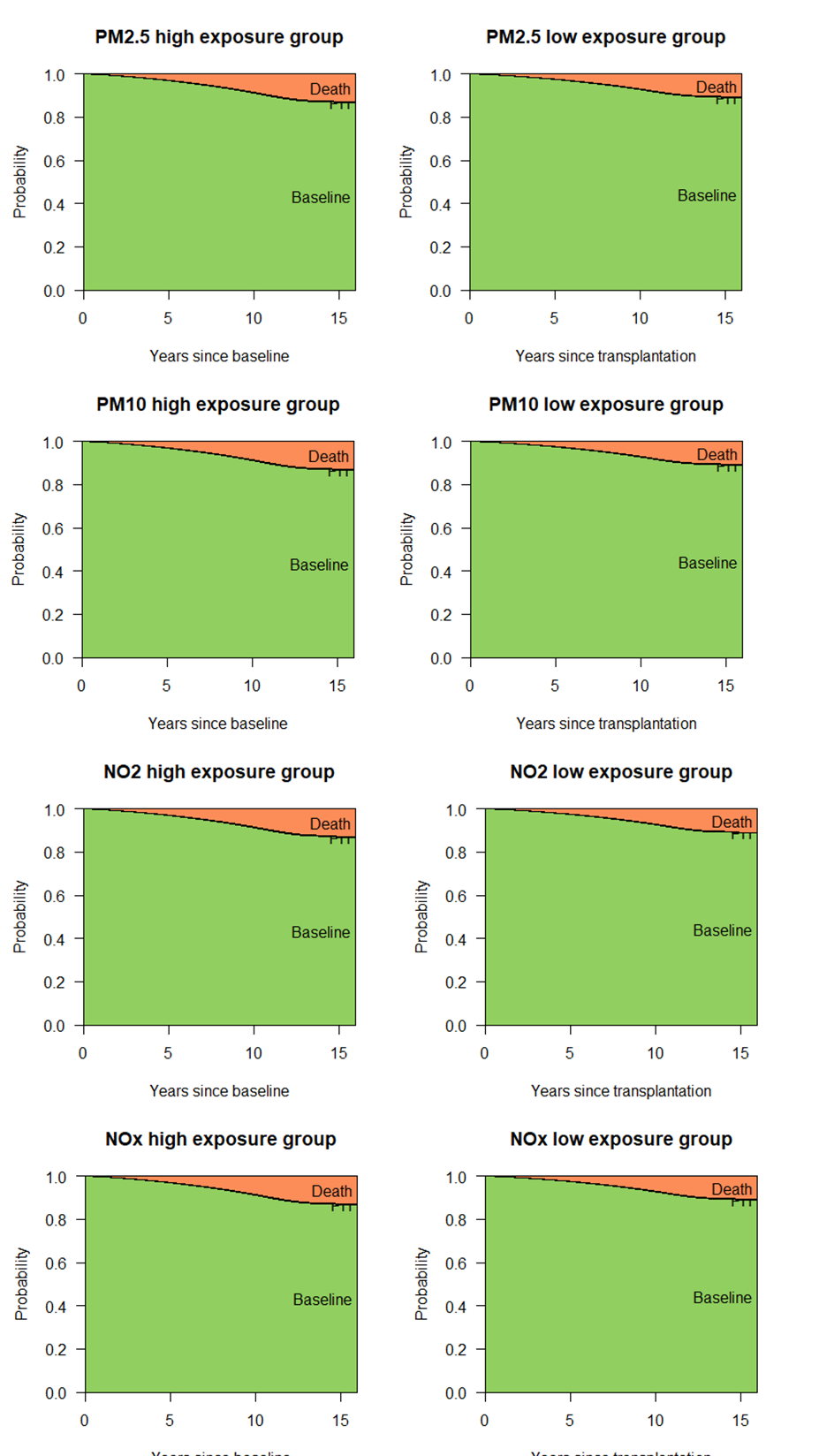


Figure S2. Estimation of the stacked transfer probability of three outcomes for participants exposed to high and low air pollution. PM_2.5_, particulate matter with a diameter <2.5 µm; PM_10_, particulate matter with a diameter <10 µm; NO_2_, nitrogen dioxide; NO_x_, nitrogen oxides; PH, pulmonary hypertension.


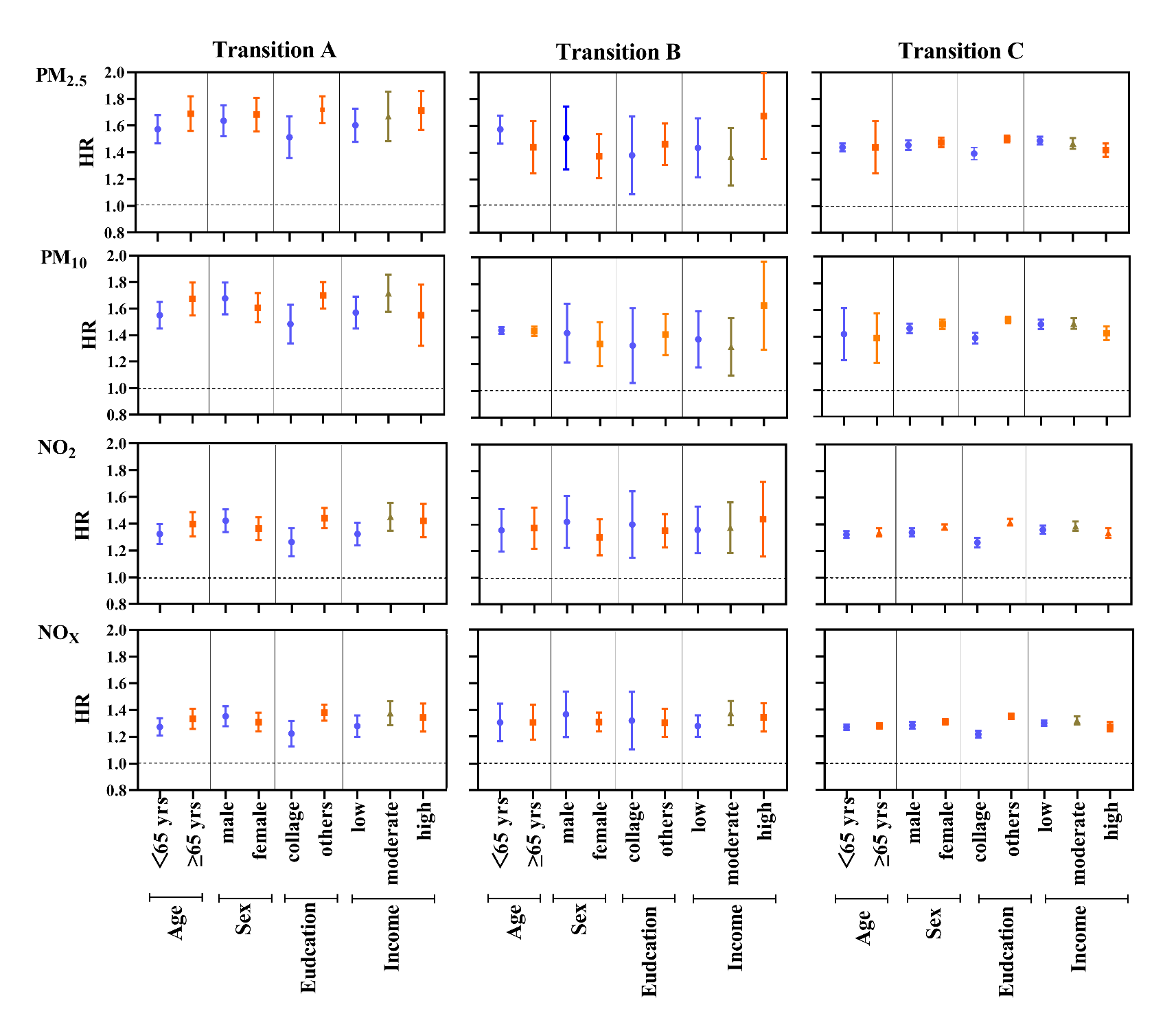


Figure S3. Associations of air pollution and three transitions in stratified analyses by sociodemographic (age, gender, education, and income). Associations are expressed per IQR increase. HR, hazard ratios, PM_2.5_, particulate matter with a diameter <2.5 µm; PM_10_, particulate matter with a diameter <10 µm; NO_2_, nitrogen dioxide; NO_x_, nitrogen oxides; PH, pulmonary hypertension.
